# Alveolar and Airway Components of the Tidal Volume in Mechanically Ventilated Dogs: An Exploratory Cross-Sectional Study

**DOI:** 10.3390/ani16040579

**Published:** 2026-02-12

**Authors:** Diego A. Portela, Pablo A. Donati, Raiane A. Moura, Francisco Medina-Bautista, Connor Cornell, Enzo Vettorato, Marta Romano, Ignacio Sández, Joaquin Araos, Pablo E. Otero

**Affiliations:** 1 Department of Comparative, Diagnostic, and Population Medicine, College of Veterinary Medicine, University of Florida, Gainesville, FL 32610, USA; 2 Department of Anesthesiology and Pain Management, Facultad de Ciencias Veterinarias, Universidad de Buenos Aires, Buenos Aires C1427CWO, Argentinapotero@fvet.uba.ar (P.E.O.); 3 Animal Medicine and Surgery Department, University of Córdoba, 14071 Córdoba, Spain; 4 Anaesthesiology Service, Veterinary Department, Biomedical and Health Sciences Faculty, Universidad Europea de Madrid, 28670 Villaviciosa de Odón, Spain; 5 Department of Clinical Sciences, College of Veterinary Medicine, Cornell University, Ithaca, NY 14850, USA; 6 Escuela de Medicina Veterinaria, Facultad Ciencias de la Vida, Universidad Andres Bello, Santiago 8370211, Chile

**Keywords:** airway dead space, alveolar tidal volume, brachycephalic breeds, canine, dogs, mechanical ventilation, tidal volume, volumetric capnography

## Abstract

Background: Tidal volume (VT) during mechanical ventilation in dogs varies considerably between individuals, but the contributions of airway dead space (VDaw) and alveolar tidal volume (VTalv) to this variability remain unclear. Methods: In this prospective, observational, exploratory study, 95 anesthetized dogs receiving volume-controlled ventilation with VT initially set at 15 mL kg^−1^ of predicted ideal body weight (IBW) were analyzed. When necessary, TV was titrated within a predetermined quasi-static driving pressure range (i.e., >6 and <10 cmH_2_O) to maintain normocapnia. Volumetric capnography was used to measure expired tidal volume (VTe), VDaw, and VTalv. The relative contributions of VDaw and VTalv to VTe variability were assessed across body sizes and breeds, including brachycephalic dogs. In addition, the effects of ideal body weight (IBW) on the variability of VTe, VDaw, and VTalv were evaluated. Results: VTalv accounted for a greater proportion of the observed VTe variability than VDaw, indicating that interindividual differences in VTalv were the primary determinant of VT variability. Brachycephalic dogs exhibited smaller VDaw but similar VTalv compared with non-brachycephalic dogs. Ideal body weight was correlated with proportionally higher VDaw and lower VTalv. Recumbency was not correlated with the observed variability in VT components. Conclusions: VTalv was the main determinant of VT variability among anesthetized dogs, and the brachycephalic breeds studied exhibited smaller VDaw but similar VTalv compared with non-brachycephalic breeds. These findings highlight the importance of considering VTalv and breed-specific airway anatomy when individualizing ventilatory settings.

## Simple Summary

Mechanical ventilation is widely used during anesthesia in dogs, but the tidal volume (VT) required can vary substantially between individuals. Tidal volume is composed of two main components: airway dead space (VDaw), which fills the conducting airways, and alveolar tidal volume (VTalv), which reaches the alveoli where gas exchange occurs. In this study, these components were measured in anesthetized dogs undergoing routine mechanical ventilation. The primary aim was to determine whether VDaw or VTalv is the main contributor to interindividual variability in VT, and how these components relate to body weight and breed. The results showed that VTalv accounted for most of the variability in VT, whereas VDaw contributed less. Brachycephalic breeds exhibited smaller VDaw but similar VTalv compared with non-brachycephalic breeds. These findings highlight the importance of considering alveolar volume and breed-specific airway anatomy when setting VT, potentially improving individualized ventilation strategies in dogs.

## 1. Introduction

Mechanical ventilation is a cornerstone of anesthetic management, primarily aimed at optimizing pulmonary gas exchange [1]. In humans and dogs, intraoperative ventilatory strategies significantly influence the incidence of postoperative pulmonary complications [2,3]. Among the key mechanical ventilation parameters, optimal titration of tidal volume (VT) is critical for ensuring adequate alveolar ventilation while minimizing ventilator-induced lung injury [4].

In veterinary medicine, VT setting in small animals has traditionally been recommended within the range of 10–20 mL kg^−1^, with several studies suggesting approximately 15 mL kg^−1^ as a standard for dogs, regardless of breed or body conformation [5,6,7]. However, dogs exhibit remarkable morphometric diversity [8], raising concerns regarding the suitability of indexing VT only to body weight across individuals of different breeds [5,9,10]. Recently, Donati et al. [11] reported that a driving pressure–guided titration of VT, as opposed to a fixed weight–based selection, resulted in improved respiratory compliance and led to an extensive range of VT values between dogs of different morphometric conformation (i.e., 11–24 mL kg^−1^).

Tidal volume can be divided into the airway dead space (VDaw) and alveolar tidal volume (VTalv) [12,13]. The VDaw corresponds to the portion of the VT that fills the conducting airways and does not reach the alveoli, whereas VTalv represents the fraction of the VT reaching the alveoli. In healthy lungs, a small proportion of VTalv may ventilate non-perfused alveoli, and this component is defined as the alveolar dead space (VDalv) [12]. The VTalv can be estimated by subtracting the volume representing the airway (i.e., VDaw) from the VT using volumetric capnography (VCap).

Volumetric capnography is a non-invasive monitoring technique that provides continuous, breath-by-breath analysis of expired carbon dioxide (CO_2_) as a function of exhaled volume [14]. This method allows real-time partitioning of VT into its functional components (i.e., VDaw and VTalv) on a breath-by-breath basis [12], thereby providing valuable insight into gas exchange efficiency and ventilation distribution. Despite its potential to guide individualized ventilatory strategies, its use in veterinary medicine remains scarce, likely due to the limited availability of suitable monitoring devices.

Compared to humans, dogs have relatively larger conducting airway volumes, resulting in a greater proportion of VT being wasted in VDaw, with values ranging from 6 to 9 mL kg^−1^ [6,15]. However, it remains unclear whether the large variability in VT observed in dogs is primarily attributable to differences in VDaw or in VTalv. Therefore, this observational study aimed to determine whether interindividual variability in VT among anesthetized dogs is better explained by differences in VDaw or VTalv. To address the study objective, VT components were quantified using VCap in anesthetized dogs receiving volume-controlled ventilation. The study hypothesized that interindividual variability in the VT would be influenced primarily by differences in VDaw among dogs, and that predicted ideal body weight (IBW) would show systematic associations with the functional components of VT. Given the exploratory nature of the study, the direction and magnitude of these relationships were not prespecified, allowing for the detection of potential breed- or size-related patterns in VT, VDaw and VTalv.

## 2. Materials and Methods

The design and reporting of this prospective, observational and exploratory cross-sectional study followed the STROBE guidelines [16]. The Institutional Animal Care and Use Committee, the Hospital Research Review Committee, and the Office of Clinical Trials of the College of Veterinary Medicine, University of Florida classified this study as observational and determined that a formal approved protocol was not required. Owner consent for the anonymous use of animals’ medical information for scientific purposes was obtained for all patients upon admission to the veterinary hospital.

### 2.1. Animals

A total of 100 dogs, classified as American Society of Anesthesiologists (ASA) physical status I–III, undergoing general anesthesia for non-emergency surgical procedures between August and December 2024, were included in the study. Dogs were eligible if they weighed >3 kg and had a body condition score (BCS) of 3–9/9. Exclusion criteria included dogs with hemodynamic instability, respiratory diseases, undergoing intrathoracic procedures or any condition contraindicating the use of mechanical ventilation or positive end-expiratory pressure (PEEP). Dogs undergoing laparoscopic procedures were included only if data were collected before the initiation of pneumoperitoneum. A formal sample size calculation was not performed a priori due to the exploratory nature of this observational study. Based on feasibility and expected case availability, a sample of 100 dogs was considered sufficient to describe the variability of VDaw and VTalv in the study population.

### 2.2. Anesthetic Management

Anesthetic and analgesic drugs and doses were selected by the supervising anesthesiologist based on the animal’s clinical presentation, type of surgery, demeanor, pain score, ASA physical status, and comorbidities.

Preanesthetic medications included full µ-opioid agonists, alone or combined with dexmedetomidine. Anesthesia was induced intravenously with propofol, alfaxalone, or a titrated combination of propofol and ketamine. Orotracheal intubation was performed using an appropriately sized cuffed endotracheal tube, selected according to internal diameter and length. The tube was advanced to the level of the thoracic inlet, and any portion protruding outside the oral cavity was trimmed to minimize increasing instrumental dead space. General anesthesia was maintained with isoflurane in >90% oxygen via a rebreathing system. After induction of general anesthesia, dogs were prepared for surgery in the preparation room. Thereafter, they were moved to the operating room where the surgical procedure was planned as scheduled. During the entire anesthetic procedure, animals were continuously monitored with a multiparametric monitor (Intellivue MP50, Philips, Böblingen, Germany) by a dedicated anesthetist, following the American College of Veterinary Anesthesia and Analgesia Small Animal Anesthesia and Sedation Monitoring Guidelines 2025 [17].

### 2.3. Study Protocol

Once in the operating room, dogs were positioned in the required surgical recumbency and connected to an anesthesia workstation (Carestation 620, GE Healthcare, Chicago, IL, USA). Ventilation was performed according to a predefined, sequential protocol. As standard practice in our hospital, a recruitment maneuver was performed by maintaining an inspiratory airway pressure of 30 cm H_2_O for 30 s. Subsequently, animals were ventilated using volume-controlled ventilation with an initial VT of 15 mL kg^−1^, an inspiratory pause set at 30% of inspiratory time, PEEP of 4 H_2_O, and a respiratory rate of 10 breaths minute^−1^. If required, VT was further adjusted within the predefined limits of a quasi-static driving pressure [DP_(q)_ = inspiratory plateau pressure − PEEP] > 6 and <10 cmH_2_O to maintain normocapnia, defined as an end-tidal CO_2_ (PE’CO_2_) of 30–44 mmHg [18]. Once VT was optimized, the respiratory rate (8–16 breaths minute^−1^) was adjusted if needed to maintain normocapnia. After the ventilatory settings had been established, research variables were recorded during the data collection phase, with ventilatory settings kept unchanged throughout.

The PE’CO_2_ and spirometry variables were monitored using an adult or neonatal mainstream CO_2_/flow sensor (Respironics Novametrix LLC, Wallingford, CT, USA) connected to a volumetric capnography monitor (Phillips NM3, Respironics, Wallingford, CT, USA); the neonatal sensor was used in dogs with an endotracheal tube internal diameter of 6 mm or smaller. The capnograph sensor was zeroed with room air, pneumotachograph accuracy was verified regularly using a 3 L calibration syringe at volumes ranging from 250 to 750 mL (Adjustable 3 L Calibration Syringe; A-M Systems Inc., Sequim, WA, USA), and CO_2_ sensor accuracy was confirmed against a known calibration gas (i.e., 5% CO_2_ gas) before each use. The manufacturer’s accuracy recommendations allow ±3–5% in the measured parameters.

Following a 15 min stabilization period with the final ventilatory settings kept constant, and provided that PE’CO_2_ remained stable within the target range (i.e., without upward or downward drift), a 3 min VCap recording was obtained and stored on a USB device using the monitor’s internal acquisition system. For each dog, the raw signal (approximately 30–50 breaths) was exported from the VCap monitor at a 200 Hz sampling rate (Datacoll, Respironics Novametrix LLC), transferred into a spreadsheet (Excel, Microsoft Corporation, WA, USA) via dedicated software (Flowtool, Respironics Novametrix LLC), and subsequently analyzed with a validated algorithm [19] implemented in specialized software (ICU-Lab, KleisTEK, Monopoli, BA, Italy) to derive all VCap and spirometry variables as previously described [13]. After the 3 min recording, data collection was complete, and the surgical procedure continued as originally planned; any adjustments to anesthetic or ventilatory management were made solely at the discretion of the supervising anesthesiologist.

### 2.4. Data Collection

Age, breed, sex, body weight (kg), body condition score (BCS; 1–9), ideal body weight (IBW), type of surgery, and surgical recumbency were recorded for all dogs. Body weight was obtained using a calibrated scale prior to anesthetic premedication. The IBW was calculated by adding 10% for each BCS point below 5 or subtracting 10% for each BCS point above 5, as previously described [15].

Raw data from each breath, extracted using the VCap monitor, were analyzed individually. Each breath was manually selected to determine expired tidal volume (VTe), VTalv, VDaw, dead space to tidal volume ratio (VD/VT), physiological dead space (VDphys), alveolar dead space (VDalv), inspiratory time (Tinsp), end-inspiratory pause time (Tpause), expiratory time (Texp), respiratory rate (*f*R), and PE’CO_2_ as previously described [13]. Respiratory system mechanics, including peak inspiratory pressure (PIP), plateau pressure at the end of the inspiratory pause (Pplat), PEEP, DP_(q)_, and quasistatic compliance [Cstat_(q)_], were also recorded. All volumetric and compliance variables were normalized to each dog’s predicted IBW. Each variable was calculated as the mean of approximately 10–50 consecutive breaths per dog. Data from dogs with fewer than ten adequately recorded breaths were excluded from further analysis.

### 2.5. Statistical Analysis

An initial exploratory analysis of VTe, VDaw, and VTalv using box plots was performed to identify patterns across breeds. This analysis suggested that dogs with brachycephalic and moderately brachycephalic phenotypes exhibited characteristics distinct from the remainder of the population. Accordingly, animals were divided into two groups based on breed phenotype: dogs with a brachycephalic phenotype (brachycephalic group) and dogs of all other breeds (non-brachycephalic group). Classification of brachycephalic breeds for group allocation was based on a phenotype-based breed definition reported in the brachycephalic obstructive airway syndrome literature, as described by Redondo et al. [20].

The distribution of continuous variables was assessed using the Shapiro–Wilk test. Depending on their distribution, continuous data were reported as median and 25th and 75th percentiles (Q1–Q3) or mean ± standard deviation (SD), while categorical variables were presented as absolute or relative frequencies. To evaluate the contribution of VDaw and VTalv to the variability in VTe, and to assess the impact of body recumbency on VTe, VDaw, and VTalv in both subgroups, simple linear regression models were constructed. In addition, associations between predicted IBW and the functional components of VT (i.e., weight-indexed VDaw and VTalv) were evaluated. Coefficients of determination (R^2^) were compared, with higher values indicating a greater proportion of explained variability. All models were fitted using robust standard errors.

Volumetric capnography and respiratory mechanics variables were compared between brachycephalic and non-brachycephalic groups using independent t-tests or Wilcoxon rank-sum tests, as appropriate. When statistically significant results were obtained, confidence interval values were reported to describe the effect size, and a post hoc statistical power analysis was performed. Regression models were reported both including and excluding the brachycephalic group. Additionally, VTe, VDaw, and VTalv were graphically summarized using box-and-whisker plots for breeds with at least three individuals.

Statistical analysis was performed using commercial software (STATA 13.0, StataCorp, College Station, TX, USA). GraphPad Prism Version 8.0 (GraphPad Software, Inc., San Diego, CA, USA) was used to create the plots and graphs.

## 3. Results

Of the 100 dogs initially enrolled, four were excluded due to inadequate waveform recordings, and one small dog was excluded because of an incorrect sensor selection, resulting in a total of 95 dogs included in the initial analysis. The median (Q1–Q3) body weight was 26.9 (13.8–35.9) kg. The median age was 5 (2–8) years, and the BCS was 6 (5–6). Females accounted for 53.6% of the cohort and males for 46.3%. The distribution of breeds and the types of surgical procedures performed are provided in Supplementary File S1.

**Figure 1:**
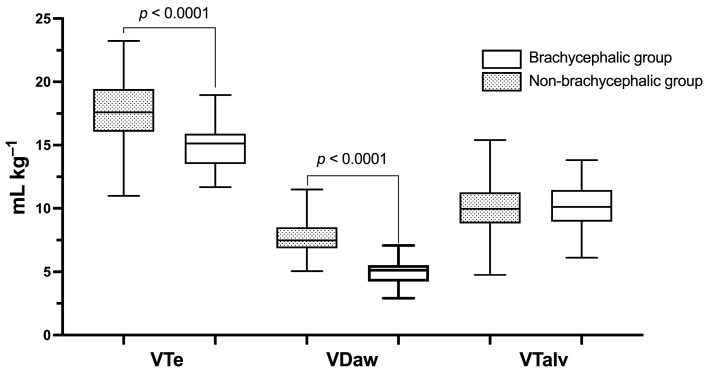
Box-and-whisker plots showing the median (central line), interquartile range (box), and range of the data (whiskers) for expired tidal volume (VTe), airway dead space (VDaw), and alveolar tidal volume (VTalv) between the non-brachycephalic group (*n* = 77) and brachycephalic group (*n* = 18). Volumes are expressed relative to the predicted ideal body weight.

**Figure 2:**
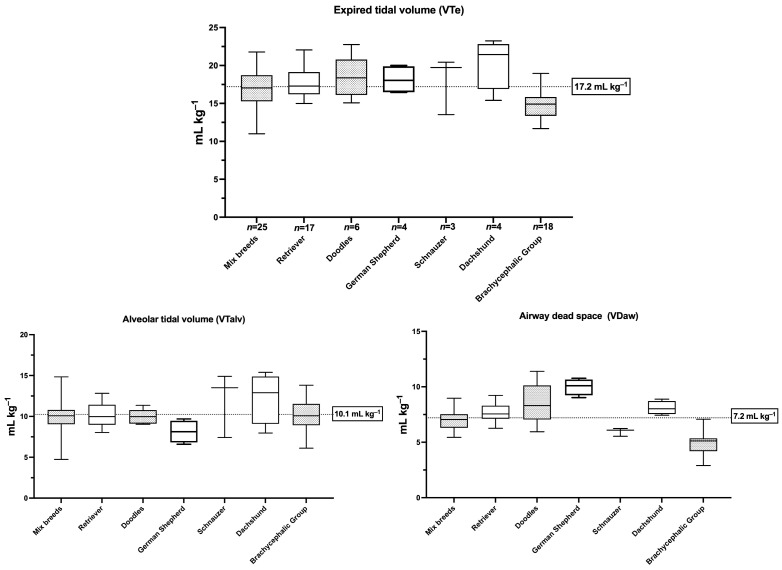
Box-and-whisker plots showing the median (central line), interquartile range (box), and range of the data (whiskers) for expired tidal volume (VTe), airway dead space (VDaw), and alveolar tidal volume (VTalv) in dogs of different breeds, including the brachycephalic group. Only breeds with more than three cases are represented. Volumes are indexed to the predicted ideal body weight. Retrievers refers to Golden and Labrador Retrievers, while Doodles includes Labradoodle and Goldendoodle breeds.

Exploratory analyses identified a group of dogs that belong to brachycephalic breeds and exhibited distinct VDaw patterns compared to the rest of the population. This group is hereafter referred to as the brachycephalic group. Consequently, subsequent analyses focused on comparisons between the brachycephalic group (*n* = 18) and the non-brachycephalic group (*n* = 77). Dogs in the brachycephalic group had significantly lower VTe and VDaw compared to the non-brachycephalic group (Table 1, Figure 1). However, no evidence of a difference was observed in VTalv between the groups (Figure 1). Summary statistics for predicted IBW, VT and its functional components, dead space measures, ventilation parameters, gas exchange, and respiratory mechanics are presented in Table 1 for reference. The effect sizes of the differences between brachycephalic and non-brachycephalic breeds, along with the post hoc statistical power analysis for data showing statistically significant differences, are presented in Supplementary Table S1. Figure 2 illustrates the partitioning of VTe into VTalv and VDaw across breed clusters with a minimum of three individuals.

When modeling VTe kg^−1^ as the dependent variable, VTalv kg^−1^ explained a larger proportion of the variance (R^2^ = 0.53) than VDaw kg^−1^ (Table 2). A similar association between VTe kg^−1^ and VTalv kg^−1^ was observed after excluding brachycephalic breeds (R^2^ = 0.64), indicating that this relationship was preserved within the non-brachycephalic subgroup.

**Figure 3:**
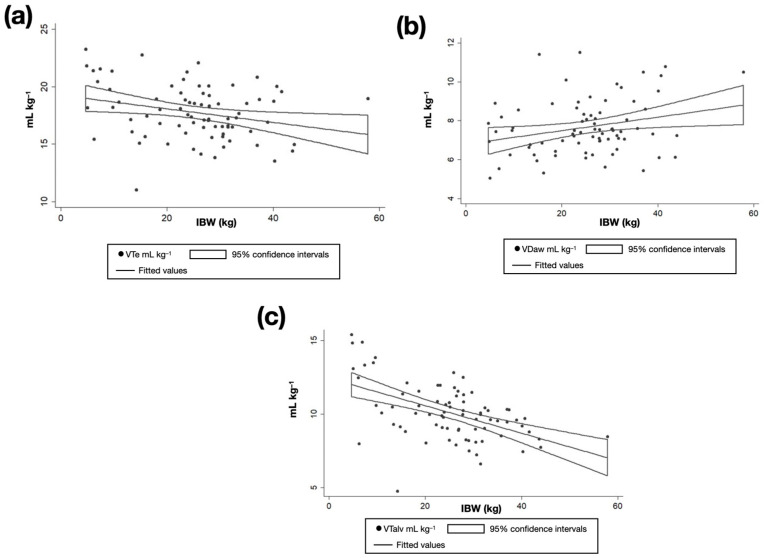
Linear regression plots showing the association between ideal body weight and (**a**) VTe, (**b**) VDaw, and (**c**) VTalv in 77 dogs without respiratory disease. Dogs with a brachycephalic phenotype (*n* = 18) were not included.

When the relationship between predicted IBW and weight-indexed VTe was analyzed, no significant association was observed in the whole cohort, indicating that VTe indexed to IBW remained relatively stable across body sizes (Figure 3, Table 3). In contrast, IBW showed a significant positive correlation with weight-indexed VDaw (coef. = 0.05, *p* = 0.001, R^2^ = 0.14) and a significant negative correlation with weight-indexed VTalv (coef. = −0.07, *p* < 0.001, R^2^ = 0.18), suggesting that increasing body size is correlated with proportionally lower VTalv and greater VDaw. After stratification by breed phenotype, these relationships remained significant in the non-brachycephalic group (Table 3). However, in the brachycephalic group, these correlations remained directionally consistent with the entire population, but did not reach statistical significance, which may reflect the limited sample size of this subgroup.

The regression analysis showed no significant effect of recumbency on ventilatory volumes in either group. In non-brachycephalic dogs, VTe, VTalv, and VDaw did not differ between dorsal, sternal, or lateral positions (all *p* > 0.27) (Supplementary Table S2). Similarly, in brachycephalic dogs, body position was not significantly associated with VTe, VTalv, or VDaw (all *p* > 0.16), and no position differed from dorsal recumbency. In all models, the coefficient of determination was low (R^2^ < 0.04), indicating that recumbency was not correlated with the observed variability in VT components (Supplementary Table S2).

## 4. Discussion

This observational, prospective, exploratory cross-sectional study suggests that in dogs mechanically ventilated using volume-controlled ventilation with VT optimized within a predefined DP_(q)_ range (i.e., >6 and <10 cmH_2_O) to maintain normocapnia, interindividual variability in VT was predominantly correlated with differences in VTalv rather than VDaw. Specifically, VTalv accounted for more than 50% of the variability in VT, whereas VDaw explained approximately 40%. Consequently, and contrary to our initial hypothesis, individual variability in VT was not primarily explained by differences in VDaw but rather by differences in VTalv. The study also identified that the brachycephalic dogs included in this study were characterized by a smaller VDaw, but a VTalv comparable to that of non-brachycephalic breeds. Moreover, under the ventilation strategy used, predicted IBW had little effect on weight-indexed VT but was associated with consistent differences in how VT was partitioned between the airway and alveolar compartments. Increasing IBW was positively correlated with VDaw and negatively correlated with VTalv, indicating that although total VT kg^−1^ remained relatively stable, larger dogs exhibited a shift in VT distribution toward the airway compartment and away from the alveolar compartment.

The exploratory analysis performed in the present study identified that the brachycephalic breeds studied had distinct ventilatory profiles, requiring significantly lower VT and higher driving pressure to achieve the target PE’CO_2_ compared to non-brachycephalic breeds. In this group of brachycephalic dogs, analysis of the functional components of VT revealed that both VDaw and VD/VT were significantly smaller than in the non-brachycephalic group, whereas VTalv remained similar to the rest of the cohort. These differences likely reflect their shortened and narrower airways, which reduce conducting airway volume while preserving alveolar structure. This indicates that, despite their unique upper airway anatomy, the alveolar volumes may be comparable to those of non-brachycephalic dogs. Additionally, dogs within the brachycephalic phenotype exhibited lower respiratory system compliance and higher peak inspiratory pressures, findings consistent with increased airway resistance and stiffer respiratory mechanics commonly reported in brachycephalic conformations [21]. These observations underscore the variability within brachycephalic phenotypes and suggest that respiratory mechanics may differ substantially among breeds sharing brachycephalic traits. The present study was not powered to formally compare brachycephalic and non-brachycephalic dogs; therefore, these observations should be considered exploratory and warrant confirmation in future, adequately powered studies.

The VDaw values observed in this study are consistent with previous reports in healthy anesthetized dogs [6,15], confirming that dogs possess relatively large conducting airways compared to humans. This difference has been attributed to species-specific allometric scaling of the respiratory tract, whereby airway volume increases disproportionately relative to alveolar surface area across different body sizes [22]. Robinson et al. [23] also reported that the lungs of dogs are larger than those of other species relative to body mass. They proposed that the versatile anatomical structure of mammalian lungs, together with thoracic conformation and gravitational effects in larger species, makes it unlikely that the same weight-indexed VT can be uniformly applied across species. It is also conceivable that differences in craniofacial and thoracic morphology among dog breeds, for instance, between brachycephalic and non-brachycephalic types, may contribute to variability in lung volume and compliance. However, this aspect was not directly assessed in the present study. While VDaw may exhibit breed-related patterns in the canine population, formal comparisons among individual breeds beyond the brachycephalic versus non-brachycephalic groups were not performed as this study was designed to evaluate interindividual variability in the components of the VT using regression-based analyses, and the limited sample size within individual breeds precluded robust breed-level comparisons.

Tidal volume selection in dogs has been widely investigated; however, no single “optimal” VT can be defined, as VT requirements depend on the physiological variable being prioritized, including gas exchange, respiratory mechanics, and lung protection. Previous studies have suggested fixed VT targets (e.g., 15 mL kg^−1^), but such recommendations are not universally applicable across breeds with marked anatomical and thoracic variability [6,7]. When a driving pressure–guided VT titration strategy is applied, VT varies as a function of respiratory system compliance, thereby revealing substantial interindividual variability [11]. In the present study, we hypothesized that this variability would primarily reflect differences in VDaw; however, our results indicate that variability in VT was driven predominantly by differences in VTalv. From a clinical perspective, these findings emphasize that fixed, weight-based VT targets may inadequately account for interindividual differences in lung volume and respiratory mechanics, potentially resulting in relative over- or underventilation in individual dogs. In contrast, when a driving pressure–guided VT titration strategy is applied, variations in VDaw and VTalv are naturally accommodated, providing a simple and physiologically sound approach for setting VT in anesthetized patients. However, this approach should not be interpreted as superior to volume-controlled ventilation, but rather as a rational method to individualize VT in a population characterized by extreme anatomical diversity.

Ideal body weight was positively correlated with VDaw and negatively with VTalv, suggesting that larger dogs may have proportionally greater airway volumes and smaller alveolar volumes per unit body mass. This pattern aligns with established allometric relationships describing respiratory system geometry, in which airway length and diameter increase faster than lung volume relative to body weight [23,24]. The weak magnitude of these correlations, however, implies that interindividual anatomical variability plays a more relevant role than body size alone. In the brachycephalic group, these relationships were not statistically significant, which may be related to the limited sample size and narrow IBW distribution of this group.

The use of a driving pressure–guided strategy to titrate VT represents a potential limitation in the interpretation of the results of this study. Adjusting VT to remain within a predefined DP_(q)_ range may preferentially influence the alveolar component of VT, as changes in respiratory system compliance predominantly affect VTalv rather than VDaw. Consequently, this approach could theoretically increase variability in VTalv relative to VDaw. Nevertheless, the application of a fixed VT (e.g., 15 mL kg^−1^) is not physiologically appropriate in a heterogeneous canine population. Dogs exhibit substantial variability in morphometric conformation and respiratory system mechanics, and VT is known to vary accordingly. Titrating VT based on DP_(q)_ allows ventilation to be individualized to each dog’s respiratory mechanics and preserves physiologically meaningful interindividual variability. In contrast, imposing a uniform VT would likely obscure true differences, result in non-physiological ventilation in many dogs, and limit meaningful assessment of the relative contributions of VTalv and VDaw. Accordingly, the findings of the present study should be interpreted as context-specific and are most directly applicable to dogs ventilated using a driving pressure–guided strategy. Extrapolation to a ventilation strategy that indexes VT solely to body weight should be made with caution, as the partitioning between alveolar and airway volumes may differ under those conditions.

Several other limitations of the present study should be acknowledged. First, although all measurements were standardized and obtained under stable anesthetic conditions, individual differences in endotracheal tube size selection or ventilatory settings may have influenced the results. Second, although brachycephalic dogs were analyzed as a distinct group, the relatively small sample size after subdivision limits the generalizability of these findings to all breeds with similar morphometric characteristics. Third, classification of dogs into the brachycephalic group was based on breed designation derived from published references [20]; however, the degree of brachycephalic conformation can vary substantially both within and across breeds. Consequently, some dogs classified as brachycephalic (e.g., Rottweilers and Yorkshire Terriers) may exhibit only mild or partial brachycephalic phenotypes, potentially introducing phenotypic overlap between groups. Fourth, our study included only a limited sample of brachycephalic breeds; therefore, our findings may not be generalizable to the entire brachycephalic canine population. Fifth, all animals were free of conditions that could alter the relationship between lung and chest wall compliance; if present, such conditions might lead to different VT responses for a given driving pressure. Finally, capnograph sensor accuracy was verified using a single calibration gas (5% CO_2_). Therefore, although accuracy at this concentration was confirmed, we cannot exclude the possibility of measurement bias at other CO_2_ concentrations.

## 5. Conclusions

In conclusion, this study suggests that in anesthetized dogs with healthy lungs ventilated using a driving pressure–guided VT titration strategy, VT exhibits substantial interindividual variability, primarily driven by differences in VTalv, likely reflecting variability in lung mechanics and thoracic conformation within breeds or phenotypic groups. Notably, although body weight has minimal impact on VT when normalized to IBW, higher body weight was associated with proportionally lower VTalv and higher VDaw. Furthermore, while VDaw contributed to total VT to a lesser extent in explaining overall interindividual variability in this cohort, breed-related differences in VDaw may still exist and may follow morphological patterns, as observed in the brachycephalic breeds included in this study. These observations are hypothesis-generating and highlight the need for future studies with adequately powered, breed-specific samples to better characterize between-breed differences and refine individualized ventilatory strategies.

## Figures and Tables

**Table 1:** Respiratory and volumetric capnography variables in 95 dogs without respiratory disease further divided into the non-brachycephalic group (*n* = 77) and brachycephalic group (*n* = 18). Values are presented as mean ± SD or median (Q1–Q3), as appropriate. All volumetric and spirometry variables are indexed to the predicted ideal body weight (IBW).

Variable	All Dogs	Non-Brachycephalic Group	Brachycephalic Group	*p* Value
IBW (kg)	25.0 [13.1–31.5]	26.4 [18.3–31.5]	11.7 [6.7–24.6]	0.002
VTe (mL kg^−1^)	17.2 ± 2.5	17.7 ± 2.4	14.9 ± 2.1	<0.001
VDaw (mL kg^−1^)	7.2 [6.1–8.1]	7.4 [6.8–8.5]	5.1 [4.2–5.1]	<0.001
VTalv (mL kg^−1^)	10.1 ± 1.9	10.1 ± 1.9	10.0 ± 1.9	0.909
VD/VT (%)	52.0 [43.0–57.0]	53.0 [48.0–59.0]	38.5 [33.9–47.0]	<0.001
VDphys (mL kg^−1^)	8.7 [7.1–10.0]	9.1 [7.9–10.4]	6.2 [4.9–8.8]	<0.001
VDalv (mL kg^−1^)	1.5 [0.8–2.0]	1.6 [0.9–2.3]	1.25 [0.6–1.7]	0.033
PE’CO_2_ (mmHg)	35.5 ± 2.6	35.4 ± 2.7	36.0 ± 2.0	0.332
Respiratory rate (bpm)	11.3 [10.0–14.0]	11.00 [9.99–14.0]	12.0 [10.00–12.7]	0.612
PIP (cmH_2_O)	13.1 ± 1.10	12.9 ± 1.0	13.9 ± 1.1	0.003
Pplat (cmH_2_O)	12.2 ± 0.9	12.0 ± 0.9	12.8 ± 0.9	0.006
PEEP (cmH_2_O)	4.0 [3.9–4.1]	4.0 [3.9–4.1]	4.0 [3.9–4.1]	0.528
DP_(q)_ (cmH_2_O)	8.1 ± 0.9	7.9 ± 0.9	8.6 ± 0.8	0.005
Cstat_(q)_ (mL cmH_2_O kg^−1^)	2.1 ± 0.4	2.2 ± 0.4	1.7 ± 0.3	<0.001
Inspiratory time (s)	1.8 [1.4–1.9]	1.8 [1.4–1.9]	1.7 [1.5–1.9]	0.468
Pause time (s)	0.4 [0.3–0.5]	0.4 [0.3–0.5]	0.4 [0.3–0.5]	0.483

VTe, expired tidal volume; VDaw, airway dead space; VTalv, alveolar tidal volume; VD/VT, physiological dead space to tidal volume ratio; VDphys, physiological dead space; VDalv, alveolar dead space; PE’CO_2_, end-expiratory carbon dioxide partial pressure; bpm, breaths per minute; PIP, peak inspiratory pressure; Pplat, plateau pressure; PEEP, positive end-expiratory pressure; DP_(q)_ (cmH_2_O), quasi-static driving pressure; Cstat_(q)_, quasi-static respiratory system compliance. *p*-values refer to comparisons between brachycephalic and non-brachycephalic groups.

**Table 2:** Simple linear regression models with VTe indexed to predicted ideal body weight (VTe, mL kg^−1^) as the dependent variable and either airway dead space volume (VDaw) or alveolar tidal volume (VTalv) as the independent variable, in a cohort of dogs without respiratory disease, analyzed overall and stratified into non-brachycephalic (*n* = 77) and brachycephalic (*n* = 18) breeds. R^2^ denotes the coefficient of determination for each model.

Group	Predictor	Coef.	SE	95% CI	*p*-Value	R^2^
All dogs	VDaw (mL kg^−1^)	0.95	0.09	0.76–1.14	<0.001	0.42
	VTalv (mL kg^−1^)	0.960	0.08	0.80–1.12	<0.001	0.53
Non-brachycephalic group	VDaw (mL kg^−1^)	0.94	0.15	0.64–1.24	<0.001	0.32
	VTalv (mL kg^−1^)	0.96	0.08	0.80–1.12	<0.001	0.64
Brachycephalic group	VDaw (mL kg^−1^)	0.67	0.38	−0.13–1.47	0.097	0.11
	VTalv (mL kg^−1^)	0.91	0.10	0.70–1.13	<0.001	0.77

VTe, expired tidal volume; VDaw, airway dead space; VTalv, alveolar tidal volume.

**Table 3:** Simple linear regression models with predicted ideal body weight as the independent variable and weight-indexed expired tidal volume (VTe), airway dead space volume (VDaw), or alveolar tidal volume (VTalv) as dependent variables in dogs without respiratory disease and stratified into non-brachycephalic breeds (*n* = 77) and brachycephalic breeds (*n* = 18). R^2^ denotes the coefficient of determination for each model.

Group	Dependent Variable	Coef.	SE	95% CI	*p* Value	R^2^
All dogs	Vte (mL kg^−1^)	−0.01	0.02	−0.06–0.03	0.589	0.004
	VDaw (mL kg^−1^)	0.05	0.01	0.02–0.08	0.001	0.14
	VTalv (mL kg^−1^)	−0.07	0.01	−0.10–−0.04	<0.001	0.18
Non-brachycephalic group	Vte (mL kg^−1^)	−0.05	0.02	−0.11–−0.001	0.043	0.07
	VDaw (mL kg^−1^)	0.03	0.01	0.001–0.06	0.038	0.07
	VTalv (mL kg^−1^)	−0.09	0.02	−0.13–−0.05	<0.001	0.26
Brachycephalic group	Vte (mL kg^−1^)	−0.02	0.04	−0.10–0.06	0.609	0.02
	VDaw (mL kg^−1^)	0.02	0.01	−0.01–0.05	0.284	0.04
	VTalv (mL kg^−1^)	−0.04	0.04	−0.12–0.04	0.336	0.06

## Data Availability

Data used for the statistical analyses are available at https://doi.org/10.5281/zenodo.18613602.

## Supplementary Materials

The following supporting information can be downloaded at: https://www.mdpi.com/article/10.3390/ani16040579/s1, Supplementary File S1. Table S1: Respiratory and volumetric capnography variables in anesthetized dogs. Data are presented for non-brachycephalic (*n* = 75) and brachycephalic (*n* = 18) dogs without respiratory disease. Values are expressed as mean ± SD or median [Q1–Q3], with 95% confidence intervals in parentheses where applicable. All volumetric and spirometry variables are indexed to predicted ideal body weight (IBW). The 95% confidence intervals are shown to indicate effect size and the power of the comparison when a statistically significant difference was observed. VTe, expired tidal volume; VDaw, airway dead space; VTalv, alveolar tidal volume; VD/VT, ratio of physiological dead space to tidal volume; VDphys, physiological dead space; PIP, peak inspiratory pressure; Pplat, plateau pressure; DP_(q)_, quasi-static driving pressure; Cstat_(q)_, quasi-static respiratory system compliance. Table S2: Linear regression models evaluating the association between body position (dorsal, sternal, and lateral recumbency) and weight-indexed expired tidal volume (VTe), airway dead space volume (VDaw), and alveolar tidal volume (VTalv) in dogs without respiratory disease and stratified in (non-brachycephalic breeds (*n* = 77) and brachycephalic breeds (*n* = 18). Reported *p* values reflect the overall model effect of recumbency, while *p* vs. *Dorsal* indicates pairwise comparisons with dorsal recumbency as the reference. R^2^ and adjusted R^2^ denote the coefficients of determination for each model.
