# Supplementary material for: Alveolar and Airway Components of the Tidal Volume in Mechanically Ventilated Dogs: An Exploratory Cross-Sectional Study
Source: Animals (Basel). 2026 Feb 12;16(4):579. doi: 10.3390/ani16040579 (PMC12937345; doi:10.3390/ani16040579)
Supplement: Supplementary file 1 [file animals-16-00579-s001.zip › Supplementary File S1.pdf]

Supplementary file S1

Breed of dogs included in the study:

From the total of 95 dogs included in the study, the most common breed was Mixed Breed (n = 25, 26%), followed by Labrador Retriever (n = 13, 13.5%), Golden Retriever (n = 8, 8.3%), and French Bulldog (n = 6, 6.3%). Other breeds included Dachshund and German Shepherd (both n = 4, 4.2%), Schnauzer (n = 3, 3.1%), American Bulldog, Chihuahua, Corgi, Labradoodle, Mastiff, and Rhodesian Ridgeback (all n = 2, 2.1%). Single dogs (n = 1, approximately 1%) were represented by Afghan Hound, Beagle, Bernese Mountain Dog, Border Collie, Boston Terrier, Cavalier King Charles Spaniel, Cocker Spaniel, Collie, Dalmatian, English Bulldog, Great Dane, Great Pyrenees, Husky, Jack Russell Terrier, Pekingese, Pointer, Poodle, Rottweiler, Weimaraner, and Yorkshire Terrier.

Type of surgeries performed on the dogs included in the study

The most frequently performed procedure was tibial plateau leveling osteotomy (TPLO; n = 21), followed by total hip replacement (THR; n = 12) and hemilaminectomy (n = 10). Other procedures included splenectomy (n = 3), tarsal arthrodesis (n = 3), mass removal from the limbs (n = 2), laparoscopic gastropexy (n = 2), brachycephalic airway surgery (BOAS; n = 2), hindlimb amputation (n = 2), arthrodesis (n = 2), and elbow arthroscopy (n = 3). Single cases were represented by forelimb amputation, cystotomy, liver lobectomy, bilateral elbow fracture repair, tail amputation, inguinal nodulectomy, tibial fracture repair, neuter, femoral ostectomy, corrective angular osteotomy, CBLO, explant arthrodesis, medial patellar luxation correction, scar revision, artificial urethral sphincter implantation, humeral fracture repair, humeral condylar fracture repair, and ulnar ostectomy. Anal sac removal procedures, including bilateral anal gland excision and anal saculectomy, were performed in three dogs.
