# Supplementary material for: Alveolar and Airway Components of the Tidal Volume in Mechanically Ventilated Dogs: An Exploratory Cross-Sectional Study
Source: Animals (Basel). 2026 Feb 12;16(4):579. doi: 10.3390/ani16040579 (PMC12937345; doi:10.3390/ani16040579)
Supplement: Supplementary file 1 [file animals-16-00579-s001.zip › Supplementary Table S1.pdf]

Supplementary Table S1 Respiratory and volumetric capnography variables in anesthetized dogs. Data are presented for non-brachycephalic (n = 75) and brachycephalic (n = 18) dogs without respiratory disease. Values are expressed as mean ± SD or median [Q1–Q3], with 95% confidence intervals in parentheses where applicable. All volumetric and spirometry variables are indexed to predicted ideal body weight (IBW). The 95% confidence intervals are shown to indicate effect size and the power of the comparison when a statistically significant difference was observed.

**Variable**	**Non-brachycephalic group**	**Brachycephalic group**	***p* value**	**Power**
IBW (kg)	26.4 [18.3–31.5]	11.7 [6.7–24.6]	0.002	0.75
VTe (mL kg^{-1})	17.7 ± 2.4 (16.9–18.5)	14.9 ± 2.1 (13.5–15.8)	< 0.001	0.99
VDaw (mL kg^{-1})	7.4 [6.8–8.5] (7.3–7.9)	5.1 [4.2-5.1] (4.3–5.3)	< 0.001	1.00
VD/VT (%)	53.0 [48.0–59.0] (50.9–55.0)	38.5[33.9–47.0] (36.5–46.1)	< 0.001	0.99
VDphys (mL kg^{-1})	9.1 [7.9–10.4] (8.9–9.7)	6.2 [4.9–8.8] (5.4–6.7)	< 0.001	1.00
VDalv (mL kg^{-1})	1.6 [0.9–2.3] (1.6–0.8)	1.25 [0.6–1.7] (1.2–0.5)	0.033	0.80
PIP (cmH_2O)	12.0 ± 0.9 (12.7–13.2)	12.8 ± 0.9 (13.3–14.4)	0.003	0.92
Pplat (cmH_2O)	12.0 ± 0.9 (11.8–12.2)	12.8 ± 0.9 (12.3–13.3)	0.006	0.82
$DP_{(q)}$ (cmH_2O)	7.9 ± 0.9 (7.8–8.2)	8.6 ±0.8 (8.2–9.1)	0.005	0.88
$Cstat_{(q)}$ (mL cmH_2O kg^{-1})	2.2 ± 0.4 (2.1–2.3)	1.7 ± 0.3 (1.6–1.8)	< 0.001	0.99

VTe, expired tidal volume; VDaw, airway dead space; VTalv, alveolar tidal volume; VD/VT, ratio of physiological dead space to tidal volume; VDphys, physiological dead space; PIP, peak inspiratory pressure; Pplat, plateau pressure; DP(q), quasi-static driving pressure; Cstat(q), quasi-static respiratory system compliance.
