# Supplementary material for: Alveolar and Airway Components of the Tidal Volume in Mechanically Ventilated Dogs: An Exploratory Cross-Sectional Study
Source: Animals (Basel). 2026 Feb 12;16(4):579. doi: 10.3390/ani16040579 (PMC12937345; doi:10.3390/ani16040579)
Supplement: Supplementary file 1 [file animals-16-00579-s001.zip › Supplementary Table S2.pdf]

Supplementary Table S2. Linear regression models evaluating the association between body position (dorsal, sternal, and lateral recumbency) and weight-indexed expired tidal volume (VTe), airway dead space volume (VDaw), and alveolar tidal volume (VTalv) in dogs without respiratory disease and stratified in (non-brachycephalic breeds (n = 77) and brachycephalic breeds (n = 18). Reported *p* values reflect the overall model effect of recumbency, while *p vs. Dorsal* indicates pairwise comparisons with dorsal recumbency as the reference. R^2 and adjusted R^2 denote the coefficients of determination for each model.

**Group**	**Parameter**	**p value**	**R^2**	**Adj R^2**	**Position**	**Mean**	**p vs. Dorsal**
Non-Brachycephalic group	VTe	0.282	0.034	0.008	Dorsal	17.63	
(n=77)					Sternal	18.87	0.136
					Lateral	17.53	0.879
	VTalv	0.273	0.034	0.008	Dorsal	10.74	—
					Sternal	11.41	0.463
					Lateral	10.02	0.435
	VDaw	0.43	0.023	-0.004	Dorsal	6.88	—
					Sternal	7.45	0.297
					Lateral	7.50	0.266
Brachycephalic group	VTe	0.18	0.202	0.095	Dorsal	14.79	—
(n=18)					Sternal	16.13	0.627
					Lateral	11.06	0.179
	VTalv	0.166	0.211	0.106	Dorsal	10.09	—
					Sternal	10.82	0.724
					Lateral	6.53	0.085
	VDaw	0.648	0.057	-0.068	Dorsal	4.70	—
					Sternal	5.31	0.551
					Lateral	4.54	0.875
